# Piperine Attenuates Lithocholic Acid-Stimulated Interleukin-8 by Suppressing Src/EGFR and Reactive Oxygen Species in Human Colorectal Cancer Cells

**DOI:** 10.3390/antiox11030530

**Published:** 2022-03-10

**Authors:** Shinan Li, Thi Thinh Nguyen, Trong Thuan Ung, Dhiraj Kumar Sah, Seon Young Park, Vinoth-Kumar Lakshmanan, Young Do Jung

**Affiliations:** 1 Research Institute of Medical Sciences, Chonnam National University Medical School, Gwangju 501-190, Korea; 156103@chonnam.edu (S.L.); thinhnt@nanogenpharma.com (T.T.N.); thuanut@nanogenpharma.com (T.T.U.); 197784@chonnam.edu (D.K.S.); drpsy@naver.com (S.Y.P.); 2 Nanogen Pharmaceutical Biotechnology Joint Stock Company, Ho Chi Minh City 70000, Vietnam; 3 Faculty of Clinical Research, Sri Ramachandra Institute of Higher Education and Research, Porur, Chennai, Tamil Nadu 600 116, India; 4 Department of Biochemistry, Chonnam National University Medical School, Seoyang Ro 264, Hwasun 58128, Korea

**Keywords:** piperine, interleukin-8, colorectal cancer, reactive oxygen species, tumor microenvironment

## Abstract

Piperine, a natural alkaloidal pungent product present in pepper plants, possesses the properties of anti-inflammatory and anti-metastasis. Lithocholic acid is a monohydroxy-5beta-cholanic acid with an alpha-hydroxy substituent at position 3; it is a secondary bile acid that plays a pivotal role in fat absorption, and has been discovered to mediate colorectal cancer (CRC) cell invasion and migration. However, the effect of piperine on angiogenesis has been poorly investigated. In the current study, we examined the role of piperine on LCA-stimulated angiogenesis by measuring interleukin-8 (IL-8) expression; moreover, we revealed the potential molecular mechanisms in CRC cells. Here, we showed that piperine inhibited LCA-stimulated endothelial EA.hy926 cell angiogenesis in a conditioned medium obtained from colorectal HCT-116 cells. Experiments with an IL-8 neutralizer showed that IL-8 present in the conditioned medium was the major angiogenic factor. Piperine inhibited LCA-stimulated ERK1/2 and AKT via the Src/EGFR-driven ROS signaling pathway in the colorectal cell line (HCT-116). Through mutagenesis and inhibitory studies, we revealed that ERK1/2 acted as an upstream signaling molecule in AP-1 activation, and AKT acted as an upstream signaling molecule in NF-κB activation, which in turn attenuated IL-8 expression. Taken together, we demonstrated that piperine blocked LCA-stimulated IL-8 expression by suppressing Src and EGFR in human CRC HCT-116 cells, thus remarkably attenuating endothelial EA.hy926 cell tube formation.

## 1. Introduction

Angiogenesis, a process involved in the formation of new blood vessels, is an essential physiological and pathological mechanism in wound healing, embryonic development, chronic inflammation, and the spread of metastatic tumors [1]. Hence, many studies have focused on the mechanisms of promotion or reduction angiogenesis [2]. As a pivotal process in tumor metastasis and progression, tumor angiogenesis is a fundamental biological mechanism for vascular network formation in the tumor microenvironment [3]. This malignant progression is determined by multiple processes, and is activated by proangiogenic factors, including vascular permeability factor (VPF), fibroblast growth factor (FGF), and interleukin-8 (IL-8) [4,5].

Interleukin-8 (IL-8), a chemoattractant cytokine, plays a major role in tumor growth, metastatic disease, and angiogenesis [5,6]. Clinical studies have indicated that abnormal concentrations of serum IL-8 are strongly correlated with metastatic stages and poor prognosis in colorectal cancer (CRC) [7]. According to our previous studies, bile acid induces endothelial cell tube formation by upregulating IL-8 expression through multiple signaling pathways [6,8]. Although the mechanism of bile acid-induced IL-8 expression in CRC remains obscure, the attenuation of IL-8 expression appears to be a possible therapeutic approach for CRC.

Piperine, well known as the main component of *Piper nigrum Linn* (black pepper), is popularly known as a seasoning food and often applied in Traditional Chinese Medicine. Some studies have demonstrated anti-inflammatory, antioxidant, anti-obesity, analgesic, and hepatoprotective properties of piperine. Moreover, anti-tumor, anti-metastatic, and anti-angiogenic properties of piperine have been reported in many studies [9,10]. In our previous study, we showed the anti-metastatic activity of piperine via the modulation of interleukin-6 expression by multiple molecular mechanisms in gastric cancer cells [11]. Based on our previous studies, and the proven anti-tumor effect of piperine, we hypothesized that piperine may inhibit LCA-stimulated IL-8 expression.

Due to the crucial importance of IL-8 in cancer growth and angiogenesis, and the anti-tumor effect of piperine, it is important to study the function of piperine on IL-8 expression, and to determine its potential role in cancer therapy. However, the inhibitory function of piperine on IL-8 expression in CRC has not been well studied. In this context, the current study addressed the inhibitory role of piperine on tumor angiogenesis via the modulation of IL-8 expression from bile acid-activated cancer cells in the tumor microenvironment, and its underlying molecular mechanism.

## 2. Materials and Methods

### 2.1. Chemicals and Reagents

Lithocholic acid (LCA) and piperine were obtained from Sigma-Aldrich (St. Louis, MO, USA). The chemicals were dissolved in dimethyl sulfoxide (DMSO) to prepare stock solutions, which were directly added to the conditioned medium (CM). The reagents were obtained from Calbiochem (San Diego, CA, USA). The Src family kinase inhibitors (PP2), LY-294002 (LY), SB-203580 (SB), and the JNK inhibitor (JNKi), BAY-11-7082 (BAY), and SN50AG-1478 (AG) were purchased from Santa Cruz Biotechnology (Santa Cruz, CA, USA). PD-98059 (PD) was purchased from New England Biolabs (Beverly, MA, USA). SR-11302 was obtained from Tocris Bioscience (Ellisville, MO, USA).

### 2.2. Cell Culture and Culture Conditions

Human colorectal cancer cell lines HCT-116, HT-29, SW-620, and SW-480 were obtained from the American Type Culture Collection (Manassas, VA, USA). The CRC cells were cultured under the conditions as described previously [6].

### 2.3. Reverse Transcription Polymerase Chain Reaction (RT-PCR) and Real-Time Quantitative Polymerase Chain Reaction (RT-qPCR)

TRIzol reagent (Invitrogen, Carlsbad, CA, USA) and M-MLV transcriptase (Promega, Madison, WI, USA) were used according to the manufacturer’s instructions. The PCR conditions were described in our previous study [12]. The RT-qPCR was performed using the Applied Biosystems™ FastStart™ SYBR Green Master Kit (Thermo Fisher, Foster City, CA, USA). The primer sequences were: IL-8 forward (accession number: NM_001354840.3, NCBI Reference Sequence), 5′-CAT ACT CCA AAC CTT TCC AC-3′; IL-8 reverse, 5′-ACT TCT CCA CAA CCC TCT GC-3′; β-actin forward (accession number: NM_000584.4, NCBI Reference Sequence), 5′-AAG CAG GAG TAT GAC GAG TCC G-3′; β-actin reverse, 5′-GCC TTC ATA CAT CTC AAG TTG G-3′. The primer sequences for RT-qPCR were: IL-8 forward (accession number: NM_001354840.3, NCBI Reference Sequence), 5′-CAT ACT CCA AAC CTT TCC AC-3′; IL-8 reverse, 5′-ACT TCT CCA CAA CCC TCT GC-3′; GAPDH forward (accession number: NM_001289745.3, NCBI Reference Sequence), 5′-TGG TAT CGT GGA AGG ACT CA-3′; GAPDH reverse, 5′-GGA TGA TGT TCT GGA GAG CC-3′.

### 2.4. Western Blot Analysis

Protein extraction solution PRO-PREP^TM^ was obtained from iNtRON Biotechnology (Jungwon, Gyeonggi, Korea). Polyvinylidene fluoride membranes and western chemiluminescent HRPV substrate were obtained from Millipore Corporation (Billerica, MA, USA). The above reagents were used according to the manufacturer’s protocol. The following antibodies from Cell Signaling Technology (Danvers, MA, USA) were used: EGFR antibody, anti-phospho-Src family (Tyr416), anti-phospho-AKT (Ser473), anti-phospho-Erk1/2, anti-p47^phox^, anti-phospho-NF-κB p65, anti-phospho-c-Fos, anti-phospho-c-Jun, anti-p44/42 MAPK (ERK1/2), anti-NF-κB p65, and anti-β-actin monoclonal antibodies. The detailed western blot steps were described previously [13].

### 2.5. ELISA (Enzyme-Linked Immunosorbent Assay) of IL-8 Secretion

The supernatants from piperine-pretreated and LCA-treated CRC cells were prepared as described previously [6]. The quantitative analysis of IL-8 secretion was performed using ELISA kit (R&D Systems, Minneapolis, MN, USA).

### 2.6. Measurement of Intracellular H_2_O_2_

ROS production levels were detected using the reactive oxygen species (ROS)-sensitive fluorophore 5- and 6- carboxyl-2′,7′-dichlorodihydro-fluorescein diacetate (DCFDA) (Grand Island, NY, USA) as described previously [14]. Briefly, after the treatment with DCFDA (5 μg/mL), the fluorescence was observed immediately using an argon laser at 488 nm, and the emission at 515 nm using a laser scanning confocal microscope.

### 2.7. NADPH Oxidase Activity Assay

The NADPH oxidase activity assay was performed following our previous method [15]. Briefly, The CRC HCT-116 cells were harvested in the NADPH lysis buffer (50 mM phosphate buffer, pH 7.0, 1 mM EGTA, 150 mM sucrose, and protease inhibitors), and centrifuged at 12,000 rpm (4 °C) for 10 min. Cell lysates with equal amount of proteins were then incubated with 10 μM diethyldithiocarbamic acid. After incubation at 37 °C for 30 min, cell lysates were then incubated with 5 μM lucigenin prepared in the NADPH lysis buffer, and 0.1 mM NADPH with or without LCA was added. After incubation for 10 min at 37 °C in dark, the NADPH oxidase complex was measured in a luminometer. The enzyme activity changes were presented as fold changes: light units/μg protein per minute.

### 2.8. Transient Transfection of Plasmid and Measurement of Promoter Luciferase Activity

FuGENE 6 and Dual Luciferase^TM^ Reporter Assay System (Promega), Madison, WI, USA were used. The IL-8 promoter–luciferase reporter construct (pGL2-IL-8), AP-1 and NF-κB luciferase reporter plasmids, dominant-negative mutants p38 MAPK (pMCL-mP38), MEK-1 (pMCL-K97M), and JNK (pMCL-TAM67) were used as in our previous study [12,16]. The transfection and co-transfection were performed as described previously [17]. Luciferase activity was determined using a luminometer (Centro XS LB960 microplate luminometer), Berthold Technologies, Bad Wildbad, Germany.

### 2.9. siRNA(Small Interfering RNA)) Transfection

The following siRNAs were used: control siRNA-A (si-Con;), c-Src siRNA (h) (si-Src), EGFR siRNA (h) (si-EGFR), AKT1 siRNA (h) (si-AKT), and p47^phox^ siRNA (h) (si- p47^phox^; Santa Cruz Biotechnology, Santa Cruz, CA, USA). siRNA oligonucleotides (50 nM) and 2 µL lipofectamine RNAi MAX (Invitrogen), Carlsbad, CA, USA were mixed with 100 µL Opti-MEM serum-free medium (Hyclone) Logan, UT, USA at room temperature for 5 min. The RNA-lipofectamine complex was added to a 6-well culture dish at 37 °C with 5% CO_2_ atmosphere for 6 h before culturing in normal growth medium.

### 2.10. Angiogenesis Assay

Conditioned medium (CM) from human CRC cells was prepared. Briefly, cells were grown to 90% confluence and incubated overnight in DMEM medium supplemented with FBS (1%). Cells were pretreated in the presence or absence of piperine for 1 h and incubated with or without 30 μM LCA for 24 h. The CRC cell supernatants were collected, centrifuged, filtered, and stored at −80 °C until use. Corning^®^ Matrigel^®^ Basement Membrane Matrix (9.1 mg/mL; Sigma-Aldrich, St. Louis, MO, USA) was loaded in a 96-well plate (60 μL/well) and incubated at 37 °C for at least 30 min. Human endothelial cells (EA.hy926) were plated (3 × 10^4^) on the prepared thin Matrigel 96-well plate with 50 μL DMEM 10% FBS media for 4 h. Subsequently, the EA.hy926 cells were incubated for 6 h with the prepared CM. IL-8 antibody (1 μg/mL; R&D Systems, Minneapolis, MN, USA) or non-specific IgG (R&D Systems, Minneapolis, MN, USA) was added to the CM before the treatment of the EA.hy926 cells with the CM. The synthesized IL-8 (1 ng/mL; Santa Cruz Biotechnology, Santa Cruz, CA, USA) along with the control CM were added to the EA.hy926 cells as the positive control for the evaluation of the IL-8 effect on endothelial cell angiogenic activity. The quantifications of nodes, junctions, branches, and segments were conducted using the Angiogenesis Analyzer (software ImageJ; http://image.bio.methods.free.fr/ImageJ/?Angiogenesis-Analyzer-for-ImageJ&lang=en&artpage=3-6#outil_sommaire_3, accessed on 25 February 2022).

### 2.11. Statistical Analysis

All values are shown as the mean ± SEM (standard error of the mean) and represent three independent experiments. The differences between two data sets were analyzed using a *t*-test. The statistically significant differences described in the text correspond to a *p* value < 0.05.

## 3. Results

### 3.1. Lithocholic Acid (LCA) Stimulates Interleukin-8 (IL-8) Expression in HCT-116 Cells

**Figure 1:**
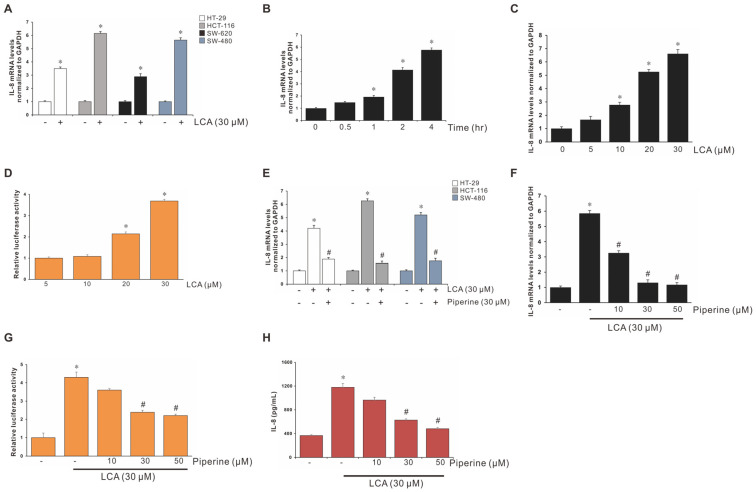
Piperine Inhibits LCA-Induced IL-8 Expression in HCT-116 Cells. (**A**) Four different human CRC cell lines were incubated with 30 μM lithocholic acid (LCA) for 4 h, followed by mRNA extraction and RT-qPCR, to determine IL-8 expression level. HCT-116 cells were incubated with 30 μΜ LCA for 0–4 h (**B**) or with 0–30 μM LCA for 4 h (**C**). (**D**) HCT-116 cells were transiently transfected with 500 ng pGL2-IL-8 promoter–reporter construct. These transfected cells were incubated with 30 μΜ LCA in a dose-dependent manner for 4 h, and the luciferase activity was measured using a luminometer. (**E**) Three different human CRC cell lines were pretreated with 30 μΜ piperine and incubated with 30 μΜ LCA for 4 h, followed by mRNA extraction and RT-qPCR to determine IL-8 expression level. (**F**) HCT-116 cells were pretreated with piperine (10, 30, and 50 μΜ) and incubated with 30 μΜ LCA for 4 h, followed by mRNA extraction and RT-qPCR to determine IL-8 expression level. (**G**) HCT-116 cells were transiently transfected with 500 ng pGL2-IL-8 promoter–reporter construct. These transfected cells were pretreated with piperine (10, 30, and 50 μΜ) and incubated with 30 μΜ LCA for 4 h, and the luciferase activity was measured using a luminometer. (**H**) HCT-116 cells were pretreated with piperine (10, 30, and 50 μΜ) and incubated with 30 μΜ LCA for 24 h, followed by ELISA assay to determine the IL-8 secretion level. Data represent the mean ± standard error of the mean (SEM) from three experimental trials. * *p* < 0.05 versus control; # *p* < 0.05 versus LCA.

The induction of interleukin-8 (IL-8) expression by LCA in human colorectal cell lines (HT-29, HCT-116, SW-620, and SW-480) was compared using reverse transcription polymerase chain reaction (RT-PCR). Treatment with LCA significantly stimulated IL-8 mRNA expression in all the colorectal cancer (CRC) cell lines tested, particularly in HCT-116 cells (Figure 1A). LCA induced IL-8 expression in a time- and dose-dependent manner (Figure 1B,C). Subsequently, the effect of LCA on transcriptional regulation was examined using the pGL2-IL-8 promoter construct. Transiently transfected HCT-116 cells with the promoter construct were incubated with various concentrations of LCA. As indicated in the Figure 1D, IL-8 promoter activity increased in a dose-dependent manner following LCA treatment.

### 3.2. Piperine Inhibits LCA-Stimulated IL-8 Upregulation in HCT-116 Cells

The effect of piperine on LCA-stimulated IL-8 expression in human CRC cell lines (HT-29, HCT-116, and SW-480) was compared using RT-PCR. As indicated in Figure 1E, piperine significantly blocked LCA-stimulated IL-8 expression in HCT-116 and SW-480 cells, whereas it was partially blocked in HT-29 cells. Combined with the results of IL-8 induction by LCA (Figure 1A), the HCT-116 cell line was used in the following experiments (Figure 1F). Subsequently, the function of piperine on LCA-stimulated IL-8 luciferase activity in CRC cells was examined using the promoter activity assay. As shown in Figure 1G, LCA-stimulated IL-8 transcription was attenuated by piperine. Similar results were observed in the enzyme-linked immunosorbent assay (ELISA; Figure 1H). The above results show that piperine suppressed LCA-stimulated IL-8 upregulation in human CRC cells.

### 3.3. Role of Activating Protein-1 (AP-1) and Nuclear Factor Kappa B (NF-κB) in LCA-Stimulated IL-8 Promoter Activity in Human CRC Cells

**Figure 2:**
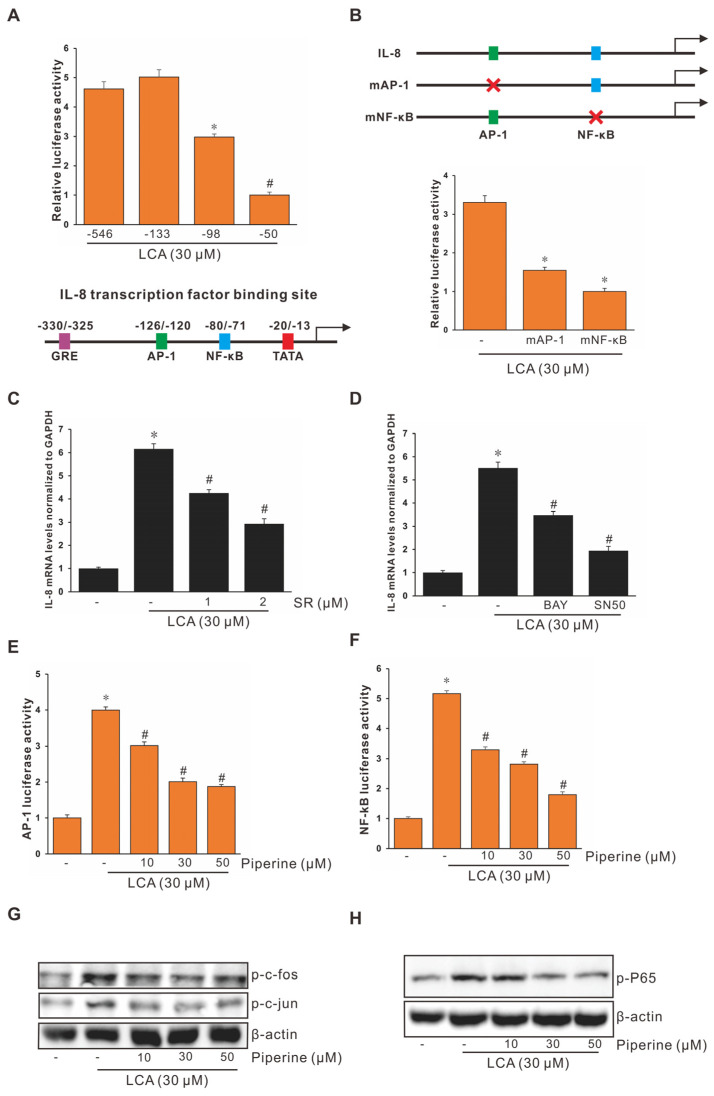
Piperine Inhibits LCA-Induced IL-8 by Suppressing the Transcriptional Activities of AP-1 and NF-κB in HCT-116 Cells. (**A**) The IL-8 promoter was sequentially deleted in the 5′-flanking region, and the promoter luciferase construct was transiently transfected into HCT-116 cells. These transfected cells were incubated with 30 μΜ LCA for 4 h, and the luciferase activity was measured using a luminometer. Data represent the mean ± SEM from three experimental trials. * *p* < 0.05 versus –133; # *p* < 0.05 versus –98. (**B**) The IL-8 promoter was mutated at the NF-κB and AP-1 binding sites (mNF-κB and mAP-1) and the promoter luciferase construct was transiently transfected into HCT-116 cells. The transfected cells were incubated with 30 μΜ LCA for 4 h, and the luciferase activity was measured using a luminometer. Data represent the mean ± SEM from three experimental trials. * *p* < 0.05 versus LCA only. HCT-116 cells were pretreated with SR-11302 (SR (**C**)), BAY-11-7082 (BAY, 20 μM (**D**)), or SN50 (50 μg/mL (**D**)) for 1 h and incubated with 30 μΜ LCA for 4 h, followed by mRNA extraction and RT-qPCR to determine IL-8 secretion level. HCT-116 cells were transiently transfected with AP-1 luciferase reporter construct (**E**) or NF-κB luciferase reporter construct (**F**). These transfected cells were pretreated with piperine (10, 30, and 50 μΜ) and incubated with 30 μΜ LCA for 4 h, and the luciferase activity was measured using a luminometer. Data represent the mean ± SEM from three experimental trials. * *p* < 0.05 versus control; # *p* < 0.05 versus IL-8. (**G**) HCT-116 cells were pretreated with piperine (10, 30, and 50 μΜ) and incubated with 30 μΜ LCA for 4 h; cell lysates were analyzed for phosphorylated c-Fos and c-Jun level using western blotting. (**H**) HCT-116 cells were pretreated with piperine (10, 30, and 50 μΜ) and incubated with 30 μΜ LCA for 4 h, cell lysates were analyzed for phosphorylated p65 level using western blotting.

As reported in our previous studies, both AP-1 and NF-κB are essential for IL-8 regulation at the transcriptional level [5,12]. Therefore, to determine the mechanism of the upregulated IL-8 expression by LCA at the transcriptional level, the DNA-binding domains of IL-8 promoter (AP-1, −126/−120; NF-κB, −80/−71) for DNA-binding molecules were checked using deletion studies. As shown in Figure 2A, a remarkable change occurred at the deletion of the upstream region of nucleotide position –98 bp as well as –50 bp, with the exception of –133 bp, after LCA treatment, indicating that the spanning positions of (–133 to –98) and (–98 to –50) exist as DNA-binding domains for LCA-inducible sites, respectively. Subsequently, site-specific mutant luciferase constructs of AP-1 and NF-κB in the IL-8 promoter were transiently transfected into human colorectal HCT-116 cells to confirm the role of AP-1 and NF-κB in LCA-stimulated IL-8 transcription. The mutant IL-8 promoter in both AP-1 and NF-κB sites showed remarkable changes in the promoter activity compared to the intact IL-8 promoter construct (Figure 2B). Based on these results, the inhibitors SR-11302 (SR, an AP-1 inhibitor) and BAY-11-7082 (BAY, an NF-κB inhibitor) were used in HCT-116 cells. As indicated in Figure 2C, SR partially blocked LCA-stimulated IL-8 expression. Piperine significantly inhibited AP-1 expression at the transcriptional level (Figure 2E), and decreased the activation of c-Fos and c-Jun molecules at the protein level (Figure 2G). Similarly, the suppression of LCA-stimulated IL-8 expression with BAY or SN50 (an NF-κB p65 nuclear translocation inhibitor) treatment is illustrated in Figure 2D. Piperine partially blocked NF-κB, both at the transcriptional and protein levels, and SN50 significantly inhibited LCA-induced IL-8 expression (Figure 2F,H).

### 3.4. Role of ERK1/2 in Suppression of LCA-Stimulated IL-8 Expression by Piperine in Human CRC Cells

**Figure 3:**
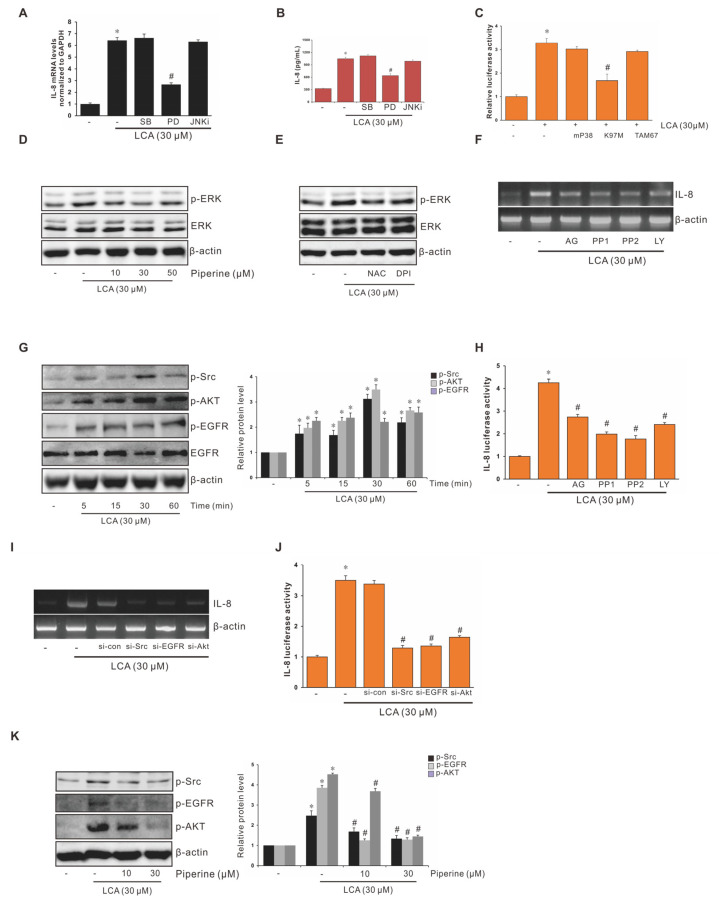
Piperine Inhibits LCA-Induced IL-8 Expression by Suppressing the Activation of ERK1/2, Src, AKT, and EGFR Signaling Pathways. (**A**) HCT-116 cells were pretreated with 30 μM concentration of SB-203580 (SB), 30 μM concentration of PD-98059 (PD), and 30 μM concentration of JNKi for 1 h, and incubated with 30 μM concentration of LCA for 4 h. Subsequently, the IL-8 mRNA level was measured through RT-qPCR. (**B**) HCT-116 cells pretreated with 30 μM SB, 30 μM PD, and 30 μM JNKi for 1 h were incubated with 30 μM LCA for 24 h followed by ELISA assay to determine the IL-8 expression level. (**C**) HCT-116 cells were transiently transfected with dominant-negative mutants of MEK-1 (K97 M), JNK (TAM67), or mutant p38 MAPK (mP38), and co-transfected with pGL2-IL-8. After incubation with 30 μM LCA for 4 h, the luciferase activity was measured using a luminometer. (**D**) HCT-116 cells pretreated with 30 μM SB, 30 μM PD, or 30 μM JNKi for 1 h were incubated with 30 μM LCA for 4 h, and cell lysates were analyzed for the phosphorylated and total ERK level using western blotting. (**E**) HCT-116 cells pretreated with 5 mM NAC or 10 μM DPI for 1 h were incubated with 30 μM LCA for 4 h, and cell lysates were analyzed for the phosphorylated and total ERK level using western blotting. (**F**) HCT-116 cells were incubated with 30 μM LCA for 0–60 min, and cell lysates were analyzed for levels of phosphorylated Src, AKT, and EGFR using western blotting. (**G**) HCT-116 cells pretreated with 10 μM AG1478 (AG), 10 μM PP1, 10 μM PP2, and 20 μM LY-294002 (LY) for 1 h were incubated with 30 μM LCA for 4 h. Subsequently, the IL-8 mRNA level was measured using RT-PCR. (**H**) HCT-116 cells pretreated with 10 μM AG, 10 μM PP1, 10 μM PP2, and 20 μM LY for 1 h were incubated with 30 μM LCA for 4 h, and the luciferase activity was measured using a luminometer. (**I**) Cells transfected with si-Con, si-Src, si-EGFR, and si-AKT were incubated with 30 μM LCA for 4 h, and IL-8 mRNA level was measured using RT-PCR. (**J**) Effects of si-Src, si-AKT, and si-EGFR on LCA-stimulated IL-8 promoter activity in CRC cells. Cells transfected with si-Con, si-Src, si-EGFR, and si-AKT were incubated with 30 μM LCA for 4 h, and the luciferase activity was measured using a luminometer. Data represent the mean ± SEM from three experimental trials. * *p* < 0.05 versus control; # *p* < 0.05 versus LCA only. (**K**) HCT-116 cells pretreated with piperine in a dose-dependent manner for 1 h were incubated with 30 μM LCA for 30 min, and cell lysates were analyzed for the phosphorylated Src, EGFR, and AKT levels using western blotting.

According to our previous study, MAPK plays an important role in bile acid-induced IL-8 expression. To examine the effect of MAPK on the mechanism underlying the suppression of LCA-stimulated IL-8 upregulation by piperine, inhibitors of ERK (PD-98059), JNK (JNKi), and p38 (SB-203580) were used in combination with LCA treatment in human CRC HCT-116 cells. As shown in Figure 3A, PD partially blocked LCA-stimulated IL-8 expression, whereas JNKi and SB did not affect IL-8 expression. Similar results were observed in a human-specific IL-8 ELISA assay (Figure 3B) and mutant studies (Figure 3C) using dominant-negative (DN) mutant plasmids mP38 (p38-DN), K97M (MEK-1-DN), and TAM67 (JNK-DN). In addition, we determined the phosphorylation levels of ERK1/2 in HCT-116 cells using western blotting. Piperine significantly blocked the phosphorylation of ERK (Figure 3D), whereas both diphenyleneiodonium chloride (DPI), an NADPH oxidase inhibitor and *N*-acetyl-l-cysteine (NAC), a reactive oxygen species (ROS) scavenger, attenuated ERK phosphorylation (Figure 3E), suggesting that piperine inhibited LCA-stimulated IL-8 expression by the regulation of ERK activation in HCT-116 cells.

### 3.5. Role of EGFR, Src, and AKT in Suppression of LCA-Stimulated Interleukin-8 (IL-8) Expression by Piperine in Human CRC Cells

To further understand the potential molecular mechanism underlying the suppression property of piperine on CRC progression, the upstream signaling pathways were identified to assess the change in phosphorylation levels of epidermal growth factor receptor (EGFR), Src, and AKT. As indicated in Figure 3F, LCA induced the phosphorylation of EGFR, Src, and AKT. Subsequent inhibitor studies confirmed that EGFR, Src, and AKT signaling pathways were involved in the suppression of LCA-stimulated IL-8 expression by piperine in HCT-116 cells, using RT-PCR (Figure 3G) and the luciferase activity assay (Figure 3H). Consistently, the results of gene silencing of Src, EGFR, and AKT using si-Src, si-EGFR, and si-AKT siRNAs, respectively, confirmed the regulatory function of Src, EGFR, and AKT in the suppression of LCA-stimulated IL-8 expression by piperine in HCT-116 cells (Figure 3I,J). As indicated in Figure 3K, piperine significantly inhibited the activation of Src, EGFR, and AKT.

### 3.6. Role of NADPH Oxidase-Derived ROS in Suppression of LCA-Stimulated IL-8 Expression by Piperine

**Figure 4:**
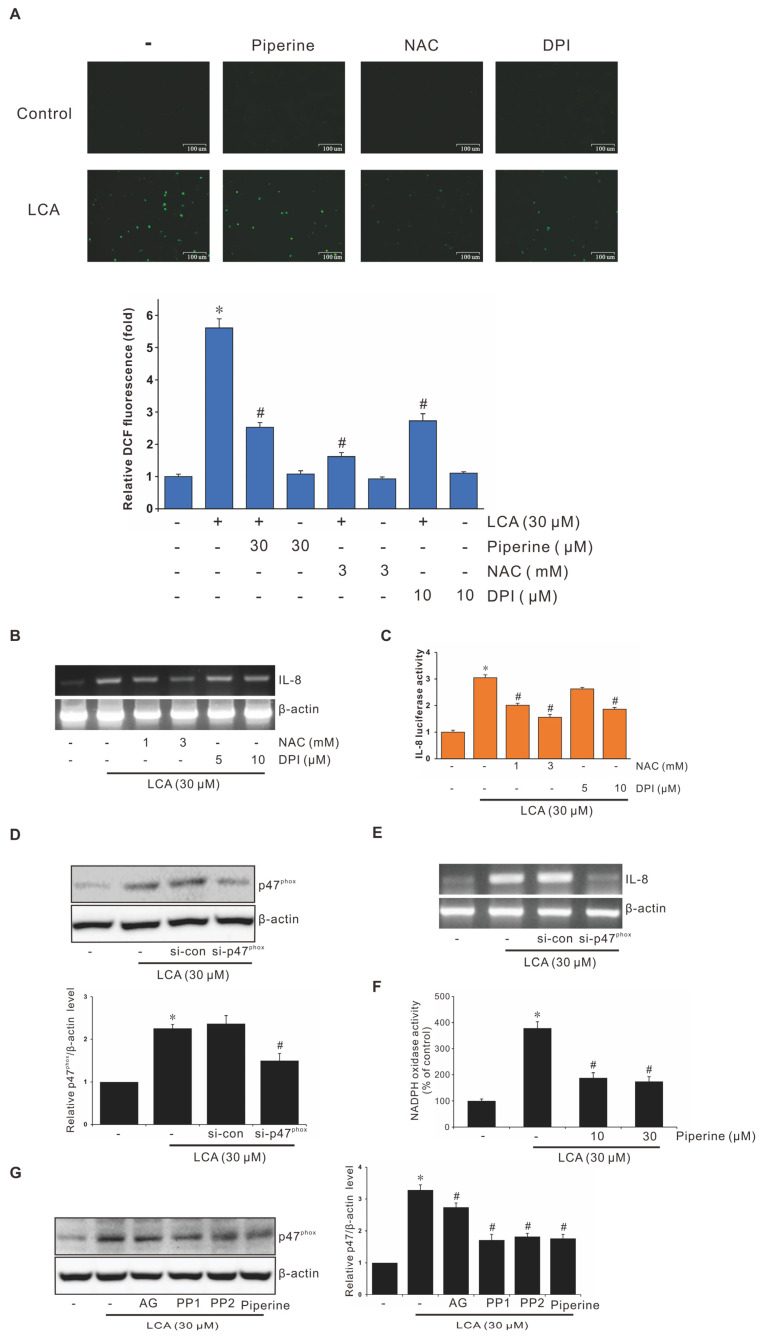
Inhibitory Role of Piperine in Activation of NADPH Oxidase-Derived Reactive Oxygen Species (ROS) during LCA-Induced IL-8 Expression in CRC Cells. (**A**) HCT-116 cells pretreated with *N*-acetyl-l-cysteine (NAC), diphenyleneiodonium chloride (DPI), or piperine for 1 h were incubated with 30 μM LCA for 10 min. Cells were then treated with 5 μg/mL of 5- and 6-carboxyl 2′,7′-dichlorodihydro-fluorescein diacetate (DCFDA) in the dark for 10 min. DCF fluorescence was imaged using a confocal laser scanning fluorescence microscope, and the statistically significant values of ROS production are presented. Scale bar: 100 μm. (**B**) Cells pretreated with NAC or DPI for 1 h were incubated with 30 μM LCA for 4 h, followed by mRNA extraction and RT-PCR to determine IL-8 expression. (**C**) HCT-116 cells were transiently transfected with 500 ng pGL2-IL-8 promoter–reporter construct. These transfected cells were pretreated with NAC or DPI for 1 h and incubated with 30 μΜ LCA for 4 h, and the luciferase activity was measured using a luminometer. Data represent the mean ± SEM from three experimental trials. * *p* < 0.05 versus control; # *p* < 0.05 versus LCA. (**D**) HCT-116 cells transfected with si-Con or si-p47^phox^ were incubated with 30 μM LCA for 30 min; cell lysates were analyzed for p47^phox^ level using western blotting. (**E**) HCT-116 cells was transfected with si-Con or si-p47^phox^ were incubated with 30 μM LCA for 4 h, and IL-8 mRNA level was measured using RT-PCR. (**F**) The NADPH oxidase activity measured by piperine pretreatment and LCA treatment. Data represent the mean ± SEM from three experimental trials. * *p* < 0.05 versus control; # *p* < 0.05 versus LCA. (**G**) HCT-116 cells pretreated with AG, PP1, PP2, or piperine for 1 h were incubated with 30 μM LCA for 4 h, and cell lysates were analyzed for p47^phox^ level using western blot analysis.

To assess the relative signals involved in suppression of ROS-driven LCA-stimulated IL-8 expression by piperine in human CRC cells, we determined ROS productions using the DCFDA assay in CRC cells pretreated with piperine and LCA. As shown in Figure 4A, LCA induced ROS production in HCT-116 cells. This induction was dramatically suppressed by piperine, NAC, and DPI. We further observed that pretreatment of human colorectal HCT-116 cells with NAC or DPI abrogated LCA-stimulated IL-8 expression at both the gene and transcription level (Figure 4B,C). As previously reported, p47^phox^ plays an important role in the activation of NADPH oxidase. Therefore, assessing the role of p47^phox^ in this study, we examined the level of NADPH oxidase subunit p47^phox^ through western blotting. As shown in Figure 4D, LCA-stimulated p47^phox^ expression was abrogated by si-p47^phox^. Additionally, the knockdown of p47^phox^ by siRNA of si-p47phox abolished LCA-stimulated IL-8 expression (Figure 4E). Piperine suppressed the LCA-stimulated NADPH oxidase activity (Figure 4F). Inhibitor studies showed that Src/EGFR were the upstream signaling molecules of p47^phox^ (Figure 4G). These results show that NADPH oxidase-derived ROS production plays an important role in the suppression of LCA-stimulated IL-8 expression via piperine.

### 3.7. Signaling Pathways Involvement in Suppression of LCA-Induced IL-8 Expression by Piperine

**Figure 5:**
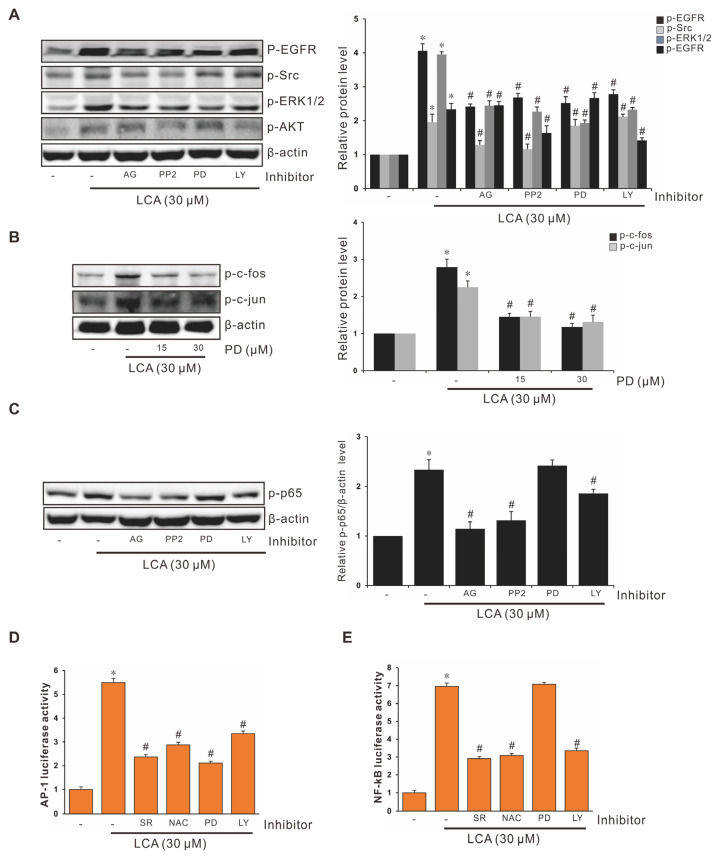
Effect of Inhibitors on Expression of EGFR, Src, ERK, and AKT in HCT-116 Cells. (**A**) HCT-116 cells pretreated with 10 μM AG1478 (AG), 10 μM PP2, 30 μM PD-98059 (PD), or 20 μM LY-294002 (LY) for 1 h were incubated with 30 μM LCA, and cell lysates were analyzed for the phosphorylation level of EGFR, Src, ERK1/2, and AKT using western blotting. (**B**) HCT-116 cells pretreated with PD for 1 h were incubated with 30 μM LCA, and cell lysates were analyzed for the phosphorylation level of c-Fos and c-Jun through western blot analysis. (**C**) HCT-116 cells pretreated with AG, PP2, PD, or LY for 1 h were treated with 30 μM LCA, and cell lysates were analyzed for the phosphorylation level of p65 using western blot analysis. HCT-116 cells were transiently transfected with AP-1 luciferase reporter construct (**D**) or NF-κB luciferase reporter construct (**E**). These transfected cells were pretreated with 2 μM SR-11302 (SR), 5 mM *N*-acetyl-l-cysteine (NAC), 30 μM PD, 20 μM LY, or 10 μM BAY-11-7082 (BAY) for 1 h and incubated with 30 μΜ LCA for 4 h, and the luciferase activity was measured using a luminometer. Data represent the mean ± SEM from three experimental trials. * *p* < 0.05 versus control; # *p* < 0.05 versus LCA.

Src/EGFR have been reported to play crucial role in a large number of signal transduction pathways, including extracellular signal transfer to the nuclei and the activation of various proteins that regulate transcription factors. To investigate their functions in the suppression of LCA-stimulated IL-8 expression by piperine, the related inhibitors were applied, and changes in protein expression and transcription were determined. As shown in Figure 5A, EGFR tyrosine kinase inhibitor (AG) treatment blocked the phosphorylation of Src/EGFR in HCT-116 cells, and similar results were observed after Src family kinase inhibitor (PP2) treatment. Phosphorylation of p-ERK1/2 was blocked by AG, PP2, PD, and the PI3K-AKT inhibitor LY-294002 (LY). Reduction of AKT phosphorylation occurred after treatment with AG, PP2, and LY, but not PD. Based on these results, the upstream role of Src/EGFR in the LCA-stimulated IL-8 expression was confirmed, and ERK1/2 and AKT acted as the downstream signaling molecules of Src/EGFR in HCT-116 cells. PD abrogated the LCA-stimulated phosphorylation of c-Fos/c-Jun (Figure 5B), indicating that ERK1/2 acted as the signaling molecule upstream of the LCA-activated AP-1 pathway. LY abrogated the LCA-stimulated phosphorylation of p65 (Figure 5C), indicating that AKT acted as the upstream signaling molecule of the LCA-activated NF-κB signaling pathway. Meanwhile, AP-1 promoter activity was blocked by inhibitors of NAC, PD, and LY, whereas NF-κB promoter activity was blocked by inhibitors of NAC and LY, but not PD (Figure 5D,E). These results suggest that AP-1 was activated by LCA through the Src/EGFR-mediated ROS-dependent ERK1/2 signaling pathway, and that NF-κB was activated by LCA through the Src/EGFR-mediated ROS-dependent AKT signaling pathway.

### 3.8. Piperine Attenuates the Conditioned Medium (CM) Dervied from LCA-Treated CRC-Induced Angiogenesis

**Figure 6:**
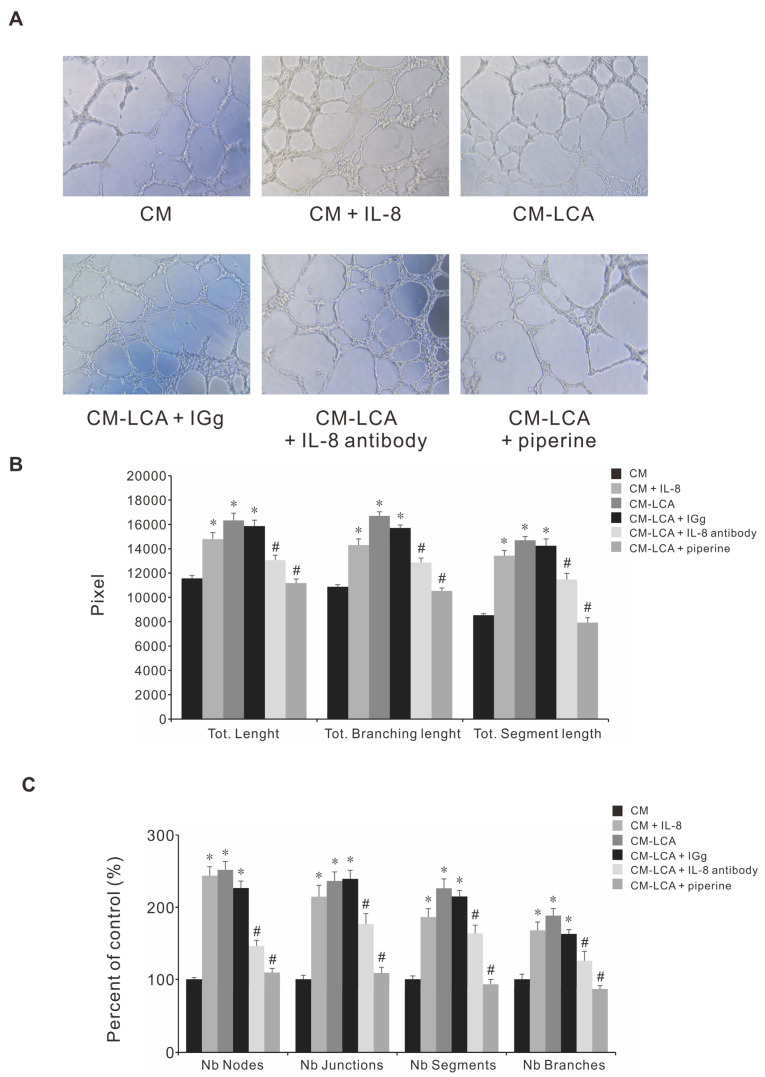
Effect of CM Obtained from the Piperine-Pretreated and LCA-Treated HCT-116 Cells on EA.hy926 for Tube Formation. EA.hy926 cells grown on a Matrigel-coated plate for 24 h were incubated with CM solutions. After 6 h, the cells were observed and counted using a Nokia microscope. (**A**) Representativeimages (10×) of the EA.hy926 tube formation. (**B**,**C**) Quantitative data of the EA. hy 926 tube formation. Data represent the mean ± SEM from three experimental trials. * *p* < 0.05 versus control; # *p* < 0.05 versus IL-8.

The anti-angiogenic property of piperine via the modulation of AKT phosphorylation has been reported [10]. We have previously demonstrated that LCA-stimulated angiogenesis occurs through IL-8 expression [6,8]. To investigate the role of piperine on LCA-stimulated angiogenesis in vitro, we performed a tube formation assay using the endothelial EA.hy926 cell line to determine the suppression property of piperine on CM with LCA-enhanced angiogenesis by attenuating IL-8 upregulation. CM derived from HCT-116 cells (CM-Control), LCA-treated HCT-116 cells (CM-LCA), and LCA-treated HCT-116 cells pretreated with piperine (CM-LCA+piperine) were prepared and used for the culturing of EA.hy926 cells. As indicated in Figure 6, the angiogenic activity significantly increased after treatment with CM-LCA compared to CM-Control, and decreased after treatment with CM-LCA+piperine compared to CM-LCA. These results suggest that piperine inhibited LCA-stimulated endothelial EA.hy926 angiogenic activity through IL-8 expression from HCT-116 cells.

## 4. Discussion

Piperine, a natural chemical compound with anti-tumor and anti-inflammatory properties, exhibits inhibitory effects, such as the suppression of NF-κB in colorectal carcinogenesis [18], as well as inhibitory effects on interleukin-8 (IL-8), interleukin-2 (IL-2), interleukin-6 (IL-6), and interleukin-1 beta (IL-1β) expression [19,20,21]. The anti-angiogenic function of piperine has been reported; piperine blocked PI3K/AKT activation, leading to the suppression of HUVEC (human umbilical vein endothelial cell) proliferation and collagen-induced angiogenesis [10]. In our study, we observed the anti-angiogenic activity of piperine using a tube formation assay on human endothelial cells (EA.hy926).

Lithocholic acid (LCA), a toxic secondary bile acid produced in the intestine, has been studied as a tumor promoter in CRC cells [22]. Kozoni et al. reported that LCA is a carcinogen that facilitates the progression of tumor by modulating the apoptotic ability of CRC cells [23]. A study by Debruyne et al. reported that LCA stimulated human CRC progression via several cancer invasion signaling pathways [24]. Our previous study has demonstrated that the metastasis-related molecule, urokinase-type plasminogen activator receptor (uPAR), was induced by LCA through the ERK-mediated AP-1 signaling pathways in CRC SW-620 cells [25]. In the current study, we demonstrated that LCA stimulates IL-8 expression by regulating the Src/EGFR-mediated ROS signaling pathway in human colorectal cells (HCT-116). Recently, the anti-carcinogenic effect of piperine via the suppression of the Wnt/β-catenin pathway in CRC HCT-116, SW-480, and DLD-1 cells was reported [26]. Hou et al. demonstrated the anti-inflammatory property of piperine; IL-8 expression was attenuated by suppressing the MAPK and NF-κB signaling pathways in lipopolysaccharide-activated CRC SW-480 and HT-29 cells [27]. Our previous study demonstrated the anti-metastatic function of piperine by blocking IL-1β-stimulated IL-6 via the downregulation of the p38 and STAT3 activation in gastric cancer TMK-1 cells [11]. In our study, the inhibitory property of piperine on LCA-stimulated IL-8-mediated angiogenesis in the tumor microenvironment was reported; moreover, additional information on the interrelated modalities was provided.

Receptor tyrosine kinases (RTKs), such as proto-oncogene tyrosine-protein kinase (Src) and epidermal growth factor receptor (EGFR), have been reported to play pivotal roles in cell migration, invasion, and angiogenesis in colorectal carcinogenesis [28,29]. Src, a non-receptor tyrosine kinase, consists of a protein tyrosine kinase (SH1) and conserved Src homology domains (SH2 and SH3). Considerably abnormal expression of Src has been observed in CRC specimens compared to normal colorectal epithelium [30]. Kumar et al. reported that soluble E-selectin promoted angiogenesis via the activation of Src and PI3K/AKT signaling pathways [31]. Advanced glycation end products enhance angiogenesis via the activation of the Src-mediated-ERK1/2 signaling pathway in human umbilical vein endothelial cells [32]. Consistently, in airway myocytes, Src signals act as the essential molecules in the regulation of IL-8, IL-6, and IL-1β expression by mediating the activation of the ERK1/2 signaling pathway. As reported by Yeh et al., Src signals are involved in oxidized phospholipid-stimulated IL-8 expression in aortic endothelial cells [33]. Moreover, IL-8 was induced by thrombin through the Src/NF-κΒ signaling pathway in a lung epithelial cell line [34]. It has also been demonstrated that Src acts as the upstream signal of polychlorinated biphenyl-stimulated IL-8 expression in human microvascular endothelial cells [35]. EGFR, the first discovered member of the ErbB (erythroblastic leukemia viral oncogene homolog) family, has been observed to be strongly regulated in normal tissues, and is aberrantly expressed in cancers [36]. It has been reported that EGFR acts as the upstream signaling molecule of the AKT pathway in the suppression of inhibitor of differentiation 3 (ID3)-stimulated IL-8 expression in glioma stem-like cells [37]. A recent study has shown that EGFR-dependent IL-8 expression by human airway epithelial cells occurs in 2,3-butanedione-induced occupational bronchiolitis obliterans [38] Consistently, Ganesan et al. reported that aberrant EGFR activation contributes to increased IL-8 expression in chronic obstructive pulmonary disease airway epithelial cells [39]. In the present study, most upstream bile acid-regulated molecules directly acted on the Src/EGFR pathway (Figure 5A). Our results are in agreement with those of Sharma et al., that previously demonstrated that LCA activates Src kinase, which regulates Bip/GRP78 expression and Golgi fragmentation in esophageal cells [40]. Akare et al. demonstrated the activation of EGFR by bile acid through the alteration of the membrane microdomains and the redistribution of cholesterol in a human CRC HCT-116 cell model [41]. Farhana et al. reported that LCA promotes colorectal carcinogenesis through EGFR activation in colorectal epithelial cells [42]. In human colorectal T84 cells, chenodeoxycholic acid stimulates cystic fibrosis transmembrane regulator (CFTR)-dependent chloride expression, which is dependent on EGFR activation [43]. Consistently, bile acid-stimulated EGFR–mitogen-activated protein kinase (EGFR–MAPK) via the modulation of the dynamic spatial distribution of minor acidic lipids in the plasma membrane of human colorectal Caco-2 cells has also been reported [44]. Src has been demonstrated to be a component of EGFR signal transduction, which independently activates EGFR [28], and was reported to be the upstream molecule of the bile acid-induced EGFR activation in rat small intestine IEC-6 cells [45]. In addition, our previous study showed that Src-dependent EGFR modulated uPAR expression in AGS cells [46].

ROS play critical roles in cancer cell progression, and are associated with multiple signaling pathways that modulate cell proliferation, invasion, and angiogenesis [47,48]. Xia et al. reported that ROS activates angiogenesis in ovarian cancer cells, using a chicken chorioallantoic membrane assay [49]. TNF-α-induced IL-8 expression via the activation of the NADPH oxidase/ROS signaling pathway has been reported by Yuan et al. [50]. In the current study, we showed that ROS participate in the regulation of LCA-stimulated IL-8, DPI (a NADPH oxidase inhibitor) and NAC (a ROS scavenger) significantly reduced the ROS production enhanced by LCA. Furthermore, regarding ROS, the angiogenesis-related molecules act as the downstream signals of NADPH oxidation in human CRC cells, which could be abrogated by piperine. Thus, our results strongly suggest that ROS act as key regulators of the suppression property of piperine on LCA-stimulated IL-8 expression in colorectal HCT-116 cells. Consistent with our results, Lee et al. discovered that the inhibition property of piperine in the migration of platelet-derived growth factor (PDGF)-BB-stimulated vascular smooth muscle cells through ROS scavenging [51]. Interestingly, piperine administration significantly suppressed NOX1 expression in the soleus muscles of mice after acute endurance exercise; similarly, piperine (10 μΜ) significantly blocked NOX1 expression in H_2_O_2_ (100 μΜ)-stimulated L6 cells [52]. In this study, we observed the upstream molecules of ROS as LCA-stimulated Src/EGFR signals in human CRC cells. Consistently, a previous study reported that Src induced ROS via NOX1 regulation in human CRC cells [53].

**Figure 7:**
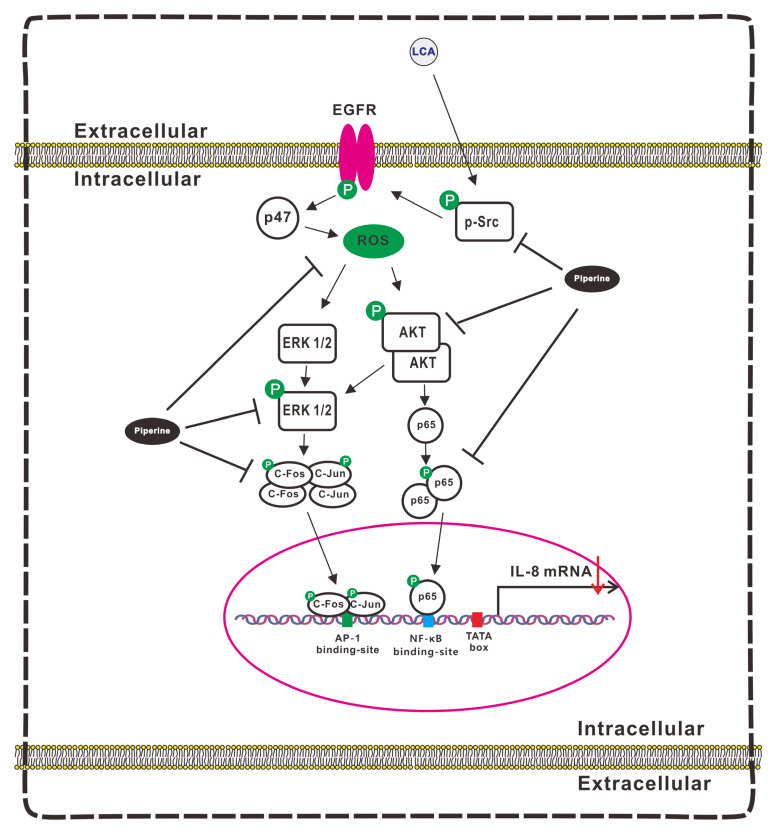
Schematic Representation of the Mechanism Underlying the Inhibitory Role of Piperine on LCA-Induced IL-8 Expression in CRC Cells and the Effect of CRC-Derived Angiogenesis in the Microenvironment. LCA induced IL-8 expression in human colorectal HCT-116 cells by increasing the transcriptional activity of AP-1 and NF-κB through the Src/EGFR-mediated ROS signaling pathways in HCT-116 cells. Piperine suppressed LCA-stimulated IL-8 expression by inhibiting the transcriptional activity of AP-1 and NF-κB and attenuating the Src/EGFR-mediated ROS-driven ERK1/2 and AKT signaling pathways. The CRC-derived IL-8 affects the endothelial EA.hy926 cell angiogenic activity in the microenvironment.

The downstream transcription factors of MAPK, such as AP-1 and NF-κB [54], participate in mediating cell invasion and migration in CRC progression [55]. AP-1 possesses two protein subunits, c-Fos and c-Jun [56], acting as a key mediator of the development of tumor metastasis [57]. Figure 2G shows that the activation levels of c-Fos and c-Jun were increased after the treatment of LCA. Figure 7 illustrates the mechanism underlying the role of LCA in colorectal progression and the inhibitory effect of piperine on LCA-stimulated CRC progression, as well as the CRC-derived IL-8 effects on the endothelial cell angiogenic activity in the microenvironment, based on the above results.

## 5. Conclusions

Our results demonstrate that piperine inhibits the CM-LCA induced endothelial angiogenic activity in the microenvironment by downregulating the IL-8 expression of CRC cells through the Src/EGFR/ROS-mediated ERK1/2 and AKT pathways. This study shows a completed series of signaling pathways associated with the role of LCA in CRC progression, and provides useful evidence for developing piperine as a new anti-cancer therapy agent for CRC progression.

## Data Availability

The data presented in this study are available in article.
